# A new insight into the evolution and functional divergence of *FRK* genes in *Pyrus bretschneideri*

**DOI:** 10.1098/rsos.171463

**Published:** 2018-07-18

**Authors:** Yunpeng Cao, Shumei Li, Yahui Han, Dandan Meng, Chunyan Jiao, Muhammad Abdullah, Dahui Li, Qing Jin, Yi Lin, Yongping Cai

**Affiliations:** 1 School of Life Sciences, Anhui Agricultural University, Hefei 230036, People's Republic of China; 2 State Key Laboratory of Tea Plant Biology and Utilization, Anhui Agricultural University, Hefei 230036, People's Republic of China

**Keywords:** FRK, pear, micro-synteny, gene duplication, expression

## Abstract

In plants, plant fructokinases (FRKs) are considered to be the main gateway of fructose metabolism as they can phosphorylate fructose to fructose-6-phosphate. Chinese white pears (*Pyrus bretschneideri*) are one of the popular fruits in the world market; sugar content is an important factor affecting the quality of the fruit. We identified 49 *FRKs* from four Rosaceae species; 20 of these sequences were from Chinese white pear. Subsequently, phylogenic relationship, gene structure and micro-collinearity were analysed. Phylogenetic and exon–intron analysis classified these *FRK*s into 10 subfamilies, and it was aimed to further reveal the variation of the gene structure and the evolutionary relationship of this gene family. Remarkably, gene expression patterns in different tissues or different development stages of the pear fruit suggested functional redundancy for *PbFRKs* derived from segmental duplication or genome-wide duplication and sub-functionalization for some of them. Additionally, *PbFRK11*, *PbFRK13* and *PbFRK16* were found to play important roles in regulating the sugar content in the fruit. Overall, this study provided important insights into the evolution of the *FRK* gene family in four Rosaceae species, and highlighted its roles in both pear tissue and fruits. Results presented here provide the appropriate candidate of *PbFRK*s that might contribute to fructose efflux in the pear fruit.

## 1. Introduction

Sucrose, fructose and glucose are the major soluble sugars in fruits [1]; however, sucrose is unstable and easily hydrolysed into glucose and fructose under the action of invertase. Hexose metabolism is a basis for photosynthetic products to enter another metabolism during fruit development stages. Though hexokinase (HXK) and fructokinase (FRK) could catalyse phosphorylation of fructose, the affinity of FRK to fructose is much higher than that of HXK [2], signifying that FRK plays an important role in fructose metabolism. Fructose could be subjected to glycolysis and oxidized pentose pathways under the catalysation of FRK via phosphorylation, which could also be used for starch synthesis. In addition, FRK can be used as a plant hexose receptor to regulate plant growth sugar signalling. Some previous studies reported that phosphorylated fructose can help to establish sink tissue [3,4], therefore, the study of fructose metabolism and *FRK* gene expression pattern in the fruit can help to expose the mechanism of sugar metabolism in the pear, and provides a scientific basis for improving fruit quality.

The *FRK* gene belongs to the phosphofructokinase B (pfkB) gene family, and the region where ATP binds to sugar is highly conserved [5]. In the higher plant tissues, the protein encoded by the *FRK* gene is present as a dimer. In the plant kingdom, the first time the *FRK* gene was isolated and identified was in tomatoes, and four family members were named *LeFRK1–4* [6–9]. *LeFRK2* and *LeFRK3* were expressed in most tissues, *LeFRK3* showed the highest expression in leaves and apical tissues, and *LeFRK4* was only expressed in stamens. Subsequently, more and more FRK was studied and reported in other plants, such as *Arabidopsis thaliana* [10–12], rice [13], poplar [14] and loquat [15] etc. In recent years, the research progress on the plant FRK gene has been accelerated. More and more plant genome sequencing has been completed, which provides more data support for the research of the *FRK* gene family. For example, the *FRK* gene family has been systematically and comprehensively studied in sugarcane and *Arabidopsis* genomes [16,17]. Subsequently, the functionally divergent *FRK* gene family members were identified in sugarcane, such as *SsFRK2* and *SsFRK7* presenting lower expression levels in response to drought stress than the other *SsFRK* genes; *SsFRK7* was under neutral selection, whereas *SsFRK2* was under strong positive selection [16].

Pear, peach, mei and strawberry, belonging to the Rosaceae family, have important economic value [18–20]. Owing to its economic prominence, the peach [21], mei [22], strawberry [23] and pear genomes [24], as well as the proteome and transcriptome of pear fruit development were sequenced. Among the main fruit varieties of China's foreign exports, the Chinese pear (*Pyrus bretschneideri*) accounts for more than 60% of the world pear production. Sugar content is an important factor affecting the taste and quality of the fruit [25]. To further expand the knowledge of FRK-catalysed phosphorylation, and understand the structure and evolutionary history of the *FRK* gene family, we investigated members of the *FRK* gene family in pear, peach, mei and strawberry. Gene structure, phylogenetic, micro-collinearity and positive selection analyses were conducted on the *FRK* gene family, and their effects on function are further discussed. Finally, the pear genome information was used and the results were verified by quantitative real-time polymerase chain reaction (qRT-PCR) experiments. The current study provides valuable information about *FRK* members, and highlights the potential candidates for further functional analysis of this important gene family, which may regulate sugar content in fruits.

## 2. Results

### 2.1. Identification of fructokinase genes

In the present study, the FRK domain (PF00294) and *A. thaliana* FRK proteins were used as queries to search against the genomes, and then 56 *FRK* genes were identified in four Rosaceae species (i.e. pear, peach, mei and strawberry). After filtering redundant genes and scanning the complete FRK domain as tested by the Pfam database, SMART database and HMMER website, 49 genes were identified as *FRK* genes for further analysis (table 1). The *FRK* genes from these four species were renamed as *PbFRK01*–*PbFRK20*, *PmFRK01*–*PbFRK15*, *PpFRK01– PbFRK08* and *FvFRK01*–*FvFRK06*, respectively. Remarkably, we found more *FRK* genes in pear than in mei, peach and strawberry, suggesting a greater expansion in the pear *FRK* genes. This phenomenon could be owing to the recent genome-wide duplication that occurred in the pear, but not in mei, peach and strawberry. However, we also noticed more *FRK* genes in mei than in peach and strawberry, despite the lack of the recent genome-wide duplication, implying that other duplication modes might play a role in the expansion of *PmFRK* genes.

**Table 1.** List of *FRK* genes identified in pear, peach, mei and strawberry.

name	gene identifier	chromosome	length (aa)	pI	mol. wt (Da)
*PbFRK11*	Pbr014370.1	chr9	386	5.25	41144.97
*PbFRK13*	Pbr009994.1	chr11	375	5.78	39589.12
*PbFRK19*	Pbr024522.1	chr17	386	5.24	41188.12
*PbFRK03*	Pbr018801.2	chr2	667	7.56	73113.69
*PbFRK08*	Pbr013899.1	chr7	358	5.77	38558.16
*PbFRK18*	Pbr012599.1	chr17	327	5.83	35075.25
*PbFRK02*	Pbr032790.1	chr1	532	6.42	57735.59
*PbFRK01*	Pbr010680.1	chr1	341	5.37	37579.94
*PbFRK17*	Pbr020141.1	chr15	370	5.38	39460.31
*PbFRK16*	Pbr005935.1	chr15	378	5.6	40214.17
*PbFRK10*	Pbr006128.1	chr8	370	4.93	39225.94
*PbFRK12*	Pbr021232.1	chr10	371	5.12	39869.69
*PbFRK07*	Pbr025309.1	chr5	371	4.96	39715.45
*PbFRK06*	Pbr032941.1	chr5	359	4.93	38271.95
*PbFRK04*	Pbr037899.1	chr3	292	6.45	31281.46
*PbFRK15*	Pbr029982.1	chr13	558	5.79	62894.34
*PbFRK09*	Pbr039958.1	chr7	369	6.18	39129.2
*PbFRK14*	Pbr014240.1	chr11	390	5.96	42116.14
*PbFRK05*	Pbr042273.1	chr3	323	5.9	34868.18
*PbFRK20*	Pbr018461.1	unanchored	430	8.03	45780.04
*FvFRK02*	mrna31198.1	chr2	361	8.5	38864.51
*FvFRK06*	mrna07640.1	unanchored	245	5.53	26436.39
*FvFRK01*	mrna21131.1	chr1	138	4.35	14842.82
*FvFRK04*	mrna18777.1	chr6	604	5.48	69110.57
*FvFRK05*	mrna17944.1	chr6	313	4.97	33625.52
*FvFRK03*	mrna21673.1	chr4	323	4.94	34772.94
*PmFRK05*	Pm014346	chr4	383	5.19	41057.04
*PmFRK07*	Pm018088	chr5	370	6.55	40190.09
*PmFRK03*	Pm007366	chr2	310	5.3	33476.53
*PmFRK09*	Pm019407	chr5	358	5.33	38510.97
*PmFRK13*	Pm022802	chr6	327	6.11	35159.53
*PmFRK08*	Pm018523	chr5	341	5.22	37548.9
*PmFRK14*	Pm030129	unanchored	341	5.22	37645.84
*PmFRK04*	Pm011272	chr3	315	9.73	33427.31
*PmFRK02*	Pm005845	chr2	374	5.48	39740.71
*PmFRK11*	Pm020464	chr6	433	5.87	46558.24
*PmFRK15*	Pm030602	unanchored	744	5.26	84046.86
*PmFRK12*	Pm021646	chr6	419	6.4	44670.5
*PmFRK10*	Pm019581	chr5	432	4.62	46075.21
*PmFRK06*	Pm017762	chr5	130	9.17	14417.05
*PmFRK01*	Pm000564	chr1	446	6.09	48771.86
*PpFRK06*	ppa004431 m	chr6	510	6.23	58052.75
*PpFRK04*	ppa006628 m	chr2	402	5.46	43182.59
*PpFRK05*	ppa007069 m	chr3	383	5.27	40971.98
*PpFRK01*	ppa008494 m	chr1	329	5.41	35299.67
*PpFRK07*	ppa008482 m	chr8	330	6.01	35501.88
*PpFRK02*	ppa003690 m	chr1	555	5.85	62608.98
*PpFRK08*	ppa026789 m	chr8	490	6.21	52629.32
*PpFRK03*	ppa008916 m	chr2	314	5.19	34153.07

**Figure 1.**
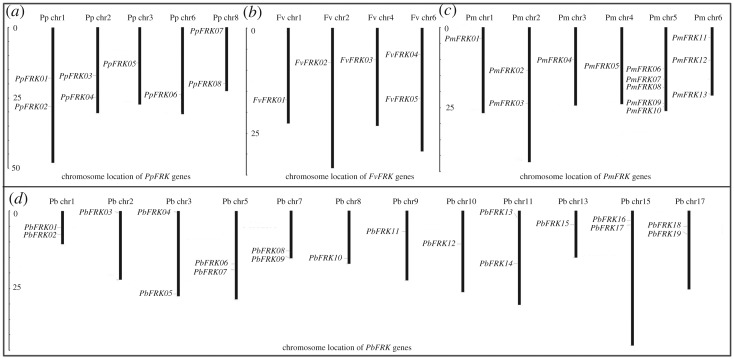
Chromosomal location of *FRK* genes among peach (*a*), strawberry (*b*), mei (*c*) and pear (*d*). According to the genomic annotation information, the physical location of individual *FRK* was mapped. The scale is indicated by mega bases (Mb).

Based on the above findings, the chromosome location of 49 *FRK* genes were randomly placed on chromosomes of the four Rosaceae species (figure 1). The *PpFRK* genes were dispersed on five chromosomes (chr 1, 2, 3, 6 and 8), the *FvFRK* genes were distributed on four chromosomes (chr 1, 2, 4 and 6) and the *PmFRK* genes mainly found on six chromosomes (chr 1, 2, 3, 4, 5 and 6), while *PbFRK* genes were distributed on twelve out of 17 chromosomes (chr 1, 2, 3, 5, 7, 8, 9, 10, 11, 13, 15 and 17). Among these genes, the longest protein was encoded by *PbFRK03*, which contains 667 amino acids (aa). However, *PmFRK06* encodes the shortest protein (130 aa). The significant difference was observed in the aa sequence length between the members. These results might be owing to the different length of the N-terminal sequence (with unknown function) or different numbers of additional insertions. As previously reported, FRKs active in plants are between 319 aa (*Solanum tuberosum* FRK, whose GenBank accession number is CAA78283) and 389 aa long (*Solanum lycopersicum* FRK3, whose GenBank accession number is NP_001234396 and includes a chloroplast transit peptide), whereas SlFRK2 (GenBank accession number: XP_004239035) is greater than 600 aa long [16,26]. Common characteristics might be shared by these proteins and active FRKs, while the additional N-terminal sequences and insertions may generate loops that might interfere with fructose binding, which has been reported by Riggs and Callis [27]. We also found that the theoretical pI values of most *FRK* proteins were less than 7, indicating that they were acidic, whereas four proteins by the other *FRK* genes (*PbFRK03*, *PbFRK20*, *PmFRK04* and *PmFRK06*) were alkaline (greater than 7). Additionally, the average molecular weight of these proteins was 42.0 kDa, with a range from 14.4 to 73.1 kDa (table 1).

### 2.2. Phylogenetic analyses

**Figure 2.**
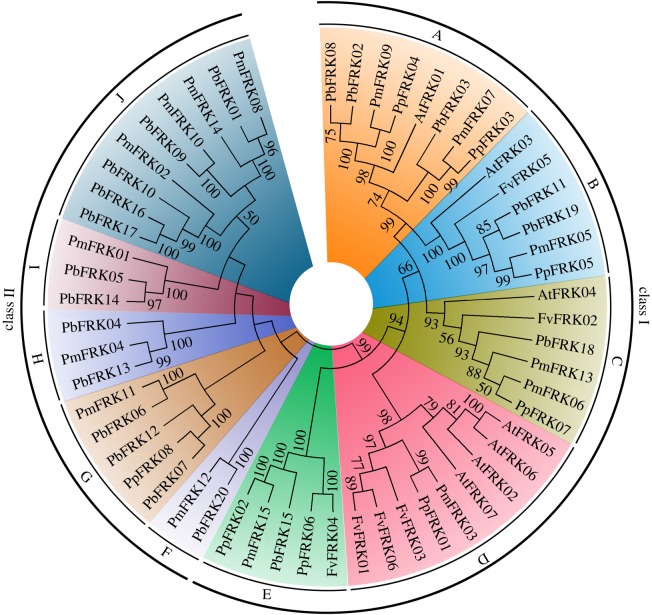
Phylogenetic analysis of all *FRK* proteins from pear, peach, mei, strawberry and *Arabidopsis*. The neighbour-joining tree of 56 *FRK* genes was generated by MEGA 5.0. The different coloured shadow marks the different subfamilies (A–J) of the *FRK* genes.

To evaluate phylogenetic relationships of *FRK* genes in these four Rosaceae species (peach, mei, strawberry and pear) and *A. thaliana*, a phylogenetic analysis was performed using the MEGA 5.0 software with the neighbour-joining (NJ) method. In *A. thaliana*, *AtFRK* genes have been described as the FRKs, which are essential for plant growth and development [28]. In our study, the NJ tree can be divided into two main classes (classes I–II) and 10 subfamilies (A–J), based on the topology and bootstrap values of the phylogenetic tree (figure 2). Remarkably, we found that class II only contained pear and mei *FRK* genes, except for *PpFRK08* (figure 2). This might be the reason why there are more pear and mei *FRK* genes than peach, strawberry and *Arabidopsis FRK* genes. At the same time, *FRK* genes from pear and mei are more closely related according to the evolutionary relationship, which is supported by a previous report showing the closer relationship between pear and mei versus pear and peach/strawberry [29].

### 2.3. Sequence feature analyses

**Figure 3.**
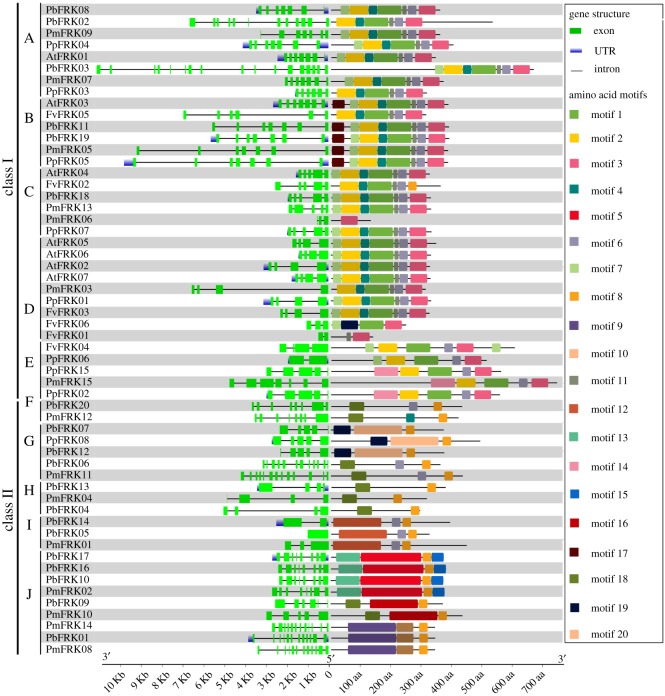
Exon–intron structure and motif compositions of 56 *FRK* genes from pear, peach, mei, strawberry and *Arabidopsis*. The relative length of gene or protein can be indicated by the scale at the bottom. Subfamilies were defined in figure 2.

In the present study, the exon–intron organization of *FRK* genes was variable in both exon size and number among the examined plant species. The exon numbers range from 1 (*PbFRK05*) to 17 (*PbFRK03)* as shown in figure 3. Furthermore, we found that the genes of each subfamily contained similar gene structures. For example, the subfamily B contained seven exons except for *FvFRK05*, which had six exons; the subfamily C contained five exons except for *PmFRK06*, which had three exons (figure 3). These results demonstrate that both exon gain and loss has occurred after the split of monocots and dicots during the evolution of the *FRK* genes, which might clarify the functional diversity of closely related genes from the *FRK* gene family (figure 3). The *FRK* paralogous or/and orthologous gene pairs' exon–intron structure was further investigated. The number of exons in eight gene pairs has changed, including *PbFRK08/PbFEK02, PbFRK03/PmFRK07, PmFEK06/PpFRK07, FvFRK06/FvFRK01, FvFRK04/PpFRK06, PmFRK15/PpFRK02, PbFRK14/PbFRK05* and *PbFRK09/PmFRK10.* Among these gene pairs, we found that *FvFRK06*, *PbFRK14* and *PbFRK09* gained one exon during the long evolutionary period, while *FvFRK01*, *PbFEK05* and *PmFRK10* lost one exon. The cause of these events may be a single intron loss or gain during the evolutionary process.

To further understand the diversity and structure characteristics of *FRK* genes, the MEME web server was used to predict the conserved domains which were shared with the 56 FRK proteins of five species (figure 3 and the electronic supplementary material, table S1). Twenty distinct motifs were found, whose distribution matched the *FRK* clustering pattern in the phylogenetic tree. The Pfam and SMART database were used to annotate each of the putative motifs. Motif 1, motif 2, motif 3, motif 5, motif 8, motif 9, motif 10, motif 16, motif 18 and motif 19 were authenticated to encode the PfkB carbohydrate kinase domain, while the other motifs’ function requires further investigation (electronic supplementary material, table S1). In addition, we found motif 1 and motif 3 mainly distributed in class I, and motif 8 distributed in class II, indicating they are necessary for the FRK proteins in these classes. To further understand the function of the different FRK proteins, we searched the gene ontology (GO) database, which provided clues on the function of 49 FRK proteins. Subsequently, we found that most *FRK* genes have some common functions, such as FRK activity, cellular metabolic process, transferase activity, biological process and molecular function (electronic supplementary material, table S2).

### 2.4. Micro-collinearity analyses

**Figure 4.**
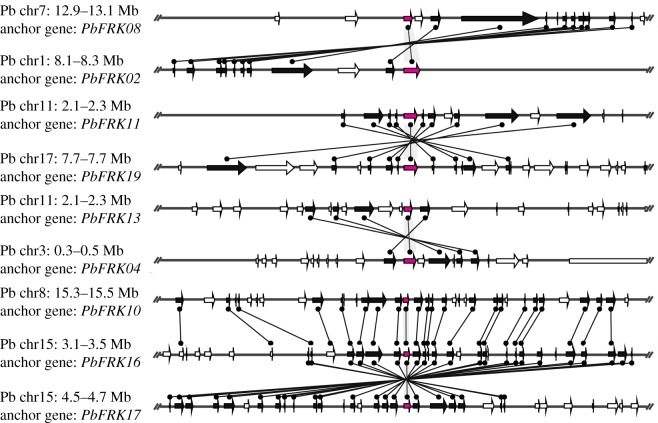
Micro-collinearity relationships of *FRK* genes in the pear. The gene names suffix is the text beside the line. The relative positions of all flanking protein-coding genes are defined by anchored *FRK* genes, highlighted in red and the homologous genes on two fragments are connected by a black line. The gene's transcriptional orientation is indicated by the triangle.

In our study, 29 paired micro-collinearity relationships were identified among four Rosaceae species. Among them, in pear and other species were found 20 micro-collinearity gene pairs (electronic supplementary material, table S3). Based on the result of MCScanX, these micro-collinearity relationships are formed by genome-wide duplication or segmental duplication. Remarkably, pear and mei micro-collinear gene pairs are much larger than those of pear and peach/strawberry; this conclusion is supported by previous reports that the relationship between pear and mei is much closer than that between pear and peach/strawberry [24,29]. At the same time, we also investigated micro-collinearity relationships among the same species. We identified six micro-collinearity gene pairs in pear, two micro-collinearity gene pairs in mei and one micro-collinearity gene pair in peach. To further understand the micro-collinearity relationships of pear *FRK* genes, the micro-collinearity diagram was generated using the MCScanX software. As shown in figure 4, all of the *FRK* gene micro-collinearity occurs in the sense direction among the pear genome.

### 2.5. Strong purifying selection for fructokinase genes in pear

The results of the previous section indicated that the expansion of the pear *FRK* gene family occurred by gene duplication. To better understand the evolutionary constraints acting on the *FRK* gene family, *K*a/*K*s values of six duplicated gene pairs were calculated among the pear *FRK* gene family. In general, Ka/Ks less than 1, equal to 1, and greater than 1 indicates purifying selection, neutral selection and positive selection, respectively. In the present study, we discovered that all the Ka/Ks values of six pear *FRK* duplicated gene pairs were less than 0.3 (electronic supplementary material, table S4). These results indicated that the *FRK* gene family had undergone a strong purifying selection, resulting in a slow evolution of the pear *FRK* gene family at the protein level. Remarkably, all the Ka/Ks ratios of 29 *FRK* duplicated gene pairs were less than 1, further indicating that this *FRK g*ene family has undergone a purifying selection during evolution (electronic supplementary material, table S4).

Previous studies have shown that overall strong purifying selection may mask some of the individual codon sites [30]. Therefore, a sliding-window analysis of Ka/Ks values was carried out for each pear *FRK* duplicated gene pair. As presented in the electronic supplementary material, figure S1, many sites or/and regions experience neutral selection, or strong negative/purifying selection. Consequently, we found that most Ka/Ks values across the coding sites/regions were much lower than 1; however, one or several distinct peaks (Ka/Ks > 1) were observed in the electronic supplementary material, figure S1. Surprisingly, the domains of *FRK* genes generally had higher Ka/Ks ratios (peaks) than the regions outside of them (valleys). A sliding-window analysis of *PbFRK13*/*PbFRK04* duplicated gene pairs revealed sites with higher Ka/Ks ratios (peaks: Ka/Ks values greater than 1) in FRK domains, implying positive selection in this site/region. Additionally, although positive selection contributes to higher values of Ka/Ks, it does not mean or ensure that the gene-average Ka/Ks ratio exceeds 1. To sum up, the above Ka/Ks values and sliding-window analyses strongly suggests that purifying selection might play a key role in the pear *FRK* gene family during evolution.

### 2.6. *Cis*-acting element analysis

*Cis*-acting elements play key roles in plant growth and development, such as determining the tissue-specific or stress-responsive expression patterns of genes. In our study, we identified the three categories of *cis*-acting elements, including plant growth and development, phytohormone responses, and biotic and abiotic stress responses (electronic supplementary material, table S5). In the promoter regions, numerous *cis*-acting elements were widespread, such as Skn-1-motif and GCN4_motif for endosperm expression, Box 4 and MRE for light responsiveness, and CAT-box and CCGTCC-box for meristem expression. Remarkably, there was a higher amount of *cis*-acting elements for plant growth and development, contrasting with the *cis*-acting elements of phytohormone responses and biotic and abiotic stress responses. These findings suggest that a number of the *FRK* gene family may have a special role in plant growth and development.

### 2.7. Expression profiles of pear fructokinase genes in different tissues

**Figure 5.**
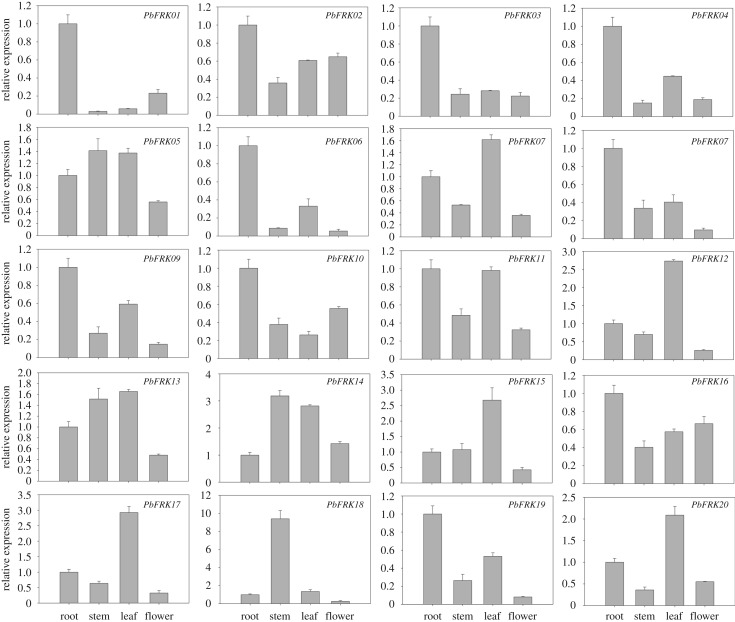
qRT-PCR expression levels of 20 *PbFRK* genes in four different tissues. Units on the *y*-axis indicate the relative expression levels. Mean values and standard deviations indicate three technical replicates derived from one bulked biological replicate.

It is well known that gene identification makes it possible to explore the potential function of gene families through gene expression analyses. To further characterize the pear *FRK* gene functions, we performed comparative gene expression analyses during different developmental stages of fruits and three different tissues (root, stem and leaf) by qRT-PCR experiments. In the present study, we found that some *PbFRK* genes were highly expressed in specific tissues or fruit development stages. For example, the expressions of nine genes (*PbFRK01*, *PbFRK02*, *PbFRK03*, *PbFRK04*, *PbFRK06*, *PbFRK08*, *PbFRK09*, *PbFRK10* and *PbFRK19*) were highly expressed in roots (*PbFRK01*, *PbFRK03*, *PbFRK04*, *PbFRK06* and *PbFRK08* were upregulated by more than 2.5-fold and *PbFRK01* showed the greatest upregulation (by more than five-fold)); *PbFRK07*, *PbFRK12*, *PbFRK13*, *PbFRK15*, *PbFRK17* and *PbFRK20* had high expression in leaves (*PbFRK12*, *PbFRK15* and *PbFRK17* were strongly upregulated (by more than 2.8-fold, 2.9-fold and 2.0-fold)); while *PbFRK05*, *PbFRK14* and *PbFRK18* peaked in stems (*PbFRK18* were highly expressed by more than 9.8-fold). These results indicated that different *PbFRK* genes may play key roles in the development of specific tissues (figure 5). Interestingly, *PbFRK11*, *PbFRK13* and *PbFRK16* were detected as downregulated during pear fruit development (electronic supplementary material, figure S2). These results showed a negative correlation with fructose levels, implying that these genes may play important roles for fructose efflux and regulate the sugar content in fruit. Moreover, some *PbFRK* genes were highly expressed at 133 days after flowering than other genes, such as *PbFRK03* and *PbFRK04,* which were upregulated more than 14-fold, and *PbFRK20,* which presented strong upregulation by more than 2000-fold, signifying that these genes may contribute to maturation in fruit development (electronic supplementary material, figure S2).

## 3. Discussion

### 3.1. Identification and phylogenetic analysis in the fructokinase gene family

It is well known that the *FRK* genes play an important role in the development of various plant organs, in fructose metabolism and in response to abiotic stresses [16,17,31,32]. Previously, the *FRK* gene family has been reported in *Arabidopsis* and *Saccharum* [16,17]. However, *FRK* genes were exclusive owing to the complex Rosaceae genome, especially in pear. In this report, we identified 49 *FRK* genes from four Rosaceae species; 20 of these sequences were from Chinese white pear, and six, eight and 15 *FRK* genes from strawberry, peach and mei, respectively. In pear, we identified a comparatively large gene family, which contained 20 *FRK* gene family members. Compared with mei, peach and strawberry, the pear genome has undergone two genome-wide duplication events [24,33]. It is believed that the expansion of the *FRK* gene family in pear might have occurred in a recent genome-wide duplication event.

Based on the topology and bootstrap values of the phylogenetic tree, all *FRK* genes were divided into two classes with 10 subfamilies (A–J). As shown in the phylogenetic tree, we observed differences between different subfamilies, suggesting that they have different biological functions. Among them, class I clustered together with the known function of the *Arabidopsis FRK* genes, whereas class II only contained the *FRK* genes from four Rosaceae species. These results indicated that we still need to explore in the future the function of these genes for different subfamilies. In addition, the pear *FRK* genes (*PbFRK16*–*PbFRK17*) from the same chromosome (no. 15) cluster together, implying the similarity of these genes. Remarkably, this duplication gene pair was not formed by tandem duplication events, because the distance between the two genes on the chromosome was greater than 200 kb [34].

### 3.2. Micro-collinearity analyses in the fructokinase gene family

Based on the micro-collinearity analyses, we identified 15761 micro-collinearity gene pairs in the pear genome to each other, 21119 gene pairs between the pear and mei genome, and 21773 gene pairs between the pear and peach genome (data not shown). These results have also been confirmed by previous reports [35], indicating strong micro-collinearity between segments of some chromosomes. Additionally, we found that a segment of chr 15 in the pear genome was thought to be micro-collinear with chr 8, and micro-collinear with chr 2 in the mei genome. The *FRK* genes *PbFRK17* and *PbFRK16* on chr 15 were micro-collinear with *PbFRK10* on chr 8; and *PbFRK17* and *PbFRK16* on chr 15 were micro-collinear with *PmFRK02* on chr 2 of mei. Interestingly, 14 micro-collinearity gene pairs were discovered between pear and mei, which were more than that between pear and peach (five), and pear and strawberry (zero), findings consistent with those of previously published manuscripts, in which the divergence of pear, mei and peach occurred after the divergence of strawberry from the common ancestor of pear, mei and peach [24,29]. At the same time, the current manuscript may provide novel resources for the study of the evolution of the *FRK* genes among different species.

### 3.3. Expression analyses of fructokinase genes in pear

Promoter elements are closely associated with potential gene functions and regulatory mechanisms, such as tissue-specific expression patterns. Previous studies have shown that *FRK* genes play an important role in fruit and seed development and play diverse functional roles in different tissues [16,28,36–38]. Chen *et al*. [16] found that most *Saccharum FRK* genes were expressed in different tissues and different developmental stages of seedlings; however, expression of several *FRK* genes was low in specific tissues. They also found that low-expressed genes in the tested tissues (such as leaf and stem) may not play the main role in fructose metabolism, such as *SsFRK2* and *SsFRK7* [16]. In the current study, we also found that some *PbFRK* genes showed low expression levels in leaves and stems, such as *PbFRK01*, which was consistent with it having fewer *cis*-acting elements for plant growth and development. Additionally, *PbFRK1*8, which had three Skn-1 motifs and one GCN4_motif, was highly expressed in fruits and stems, suggesting that this gene might primarily regulate the development of these tissues, which is consistent with tomato *SlFRK3,* which can reduce the stem xylem area and contribute to stem development [39]. Remarkably, the current study shows that almost all *PbFRK*s have low expression in roots, compared to other tissues (i.e. stem, leaf and flower). Similar results have been verified for *FRKs* from other plants, such as *Solanum lycopersicum* and Cassava, which participate in the development of roots [16,40]. The expression of *PbFRK01* and *PbFRK10* was detected by qRT-PCR, to be mainly in stems and roots, with lower expression in leaves. This result was similar to that of the *FRK1* and *FRK2* genes in maize [41], indicating that these genes may play an important role in the metabolism of sink tissues. In general, the expression pattern of duplicated gene pairs should be similar [42]. In our study, we found that some of the paralogous gene pairs (*PbFRK*02/-*08*, *PbFRK11*/-*19* and *PbFRK10*/-*16*) have similar expression patterns, indicating that these genes were found to be non-divergent after duplication and might retain redundant functions in regulating tissue development (figure 5). However, three duplicate gene pairs (*PbFRK*04/-*13*, *PbFRK10*/-*16* and *PbFRK16*/-*17*) were divergent, implying that these genes displayed sub-functionalization for some of them (figure 5).

Pear is one of the most important commercial fruit crops in the world. Sugar content is an important factor measuring fruit quality, which directly affects the flavour of the fruit. *FRK* genes have been reported to affect fructose accumulation in fruits [43,44]. Sugar transport is essential for plant development, and organisms can control sugar influx and efflux depending on supply and demand [45]. The activity and expression of FRK gradually decreased, while the sugar content gradually increased with the development of fruit [46]. As previously reported, sugar efflux and influx are regulated by related genes [47]. Here, *PbFRK11*, *PbFRK13* and *PbFRK16* were found to have a negative correlation with fructose levels during pear fruit development. These results indicated that these genes may play a key role in the balance of fructose levels during fruit development. To sum up, our study provides new ideas and directions for *FRK* gene function in the pear.

In the present study, 49 *FRK* genes from four Rosaceae species were identified; a systematic analysis including their physical locations, phylogenetic relationships, gene structure, micro-synteny and the expression patterns of pear *FRK* genes was carried out. Based on the phylogenetic analysis, all *FRK* genes were divided into two classes with 10 subfamilies. Additionally, the characteristics and composition of motif and exon/intron structure were relatively conserved in each subfamily. Our study suggested that the vast majority of *PbFRK* genes were expanded by segmental duplication or genome-wide duplication. Subsequently, these genes were found to undergo strong purifying selection, indicating their evolution at the protein level was slow. Furthermore, the expression patterns of *PbFRK* genes identified that these genes play key roles during pear fruit development.

## 4. Material and methods

### 4.1. Data sources and sequence retrieval

First of all, the peach [21], mei [22], strawberry [23] and pear [24] genome files were obtained from the GigaDB (http://gigadb.org/site/index), Phytozome database (https://phytozome.jgi.doe.gov/pz/portal.html) and GDR database (http://www.rosaceae.org/), respectively. The *A. thaliana FRK* genes were obtained from Riggs *et al*. [17] and were downloaded from the TAIR database (http://www.arabidopsis.org/). The seed of FRK domain PF00294 were obtained from the Pfam database (http://pfam.xfam.org/) [48]. Subsequently, the *FRK* genes were searched against the fully sequenced genome of four Rosaceae species (peach, strawberry, mei and pear) using the HMMER3 software package (*E* value less than 1 × 10^−10^) [49] and the BlastP software. After filtering, according to the results of further analysis for sequence identification numbers, chromosomal location and sequence alignment, we deleted redundant protein sequences. At the same time, to verify the putative *FRK* genes, all the proteins for the presence of FRK domain PF00294 were taken from the SMART [50], Pfam [48] and HMMER website (https://www.ebi.ac.uk/Tools/hmmer/search/hmmscan).

### 4.2. Phylogenic and gene structure analysis

Coding sequence alignments for 56 FRK proteins from *A. thaliana*, peach, mei, strawberry and pear were conducted using the MUSCLE program with default settings in the MEGA 5.0 software [51]. Based on alignments, the NJ trees were generated by the MEGA 5.0 software with bootstrap 1000. To identify the exon–intron organization, we compared the CDS sequence with its corresponding DNA sequence and submitted the GSDS website [52] for visualization. The conserved motifs analysis of the FRK proteins was performed by using the MEME software according to previous studies [29,30,42,53]. The GO annotations of the FRK proteins were analysed using the Blast2GO software according to previous studies [54].

### 4.3. Chromosome location analysis

According to the publicly available annotation information of chromosome locations in the GigaDB database (http://gigadb.org/site/index) and the Phytozome database (http://www.phytozome.net), chromosomal location images of *FRK* genes were drawn by the TBtools software.

### 4.4. *Cis*-acting elements analysis

To analyse the *cis*-acting elements, the 2000 bp upstream genomic DNA sequences with the initiation code (ATG) of *FRK* genes were obtained from the pear, mei, peach and strawberry genome, respectively. The presence of different *cis*-acting elements was determined by using the PlantCARE website (http://bioinformatics.psb.ugent.be/webtools/plantcare/html/) [55].

### 4.5. Micro-synteny analysis

First of all, we downloaded the whole-genome sequences of the four Rosaceae species to our local server. Subsequently, the MCScanX software [56] was used for detecting the collinear blocks relation of each pair of species. The procedure in ColinearScan was used to evaluate the resulting collinearity chains with an *E-*value of less than 1 × 10^−10^. The CODEML program of PAML [57] was used to calculate the non-synonymous substitution rate (Ka) and the synonymous substitution rate (Ks). The selection pressure in evolution was detected by using the values of Ka/Ks between each gene pair.

### 4.6. RNA extraction and quantitative real-time polymerase chain reaction analysis

RNAisomate for Plant Tissue Kit (Takara, Dalian, China) was used to extract total RNA from each frozen tissue. RNA (1 μg) was used as the template to synthesize first-strand cDNAs using the PrimeScript™ RT reagent Kit with the gDNA Eraser (Takara, Dalian, China). qRT-PCR was performed using SYBR® Premix Ex Taq™ II (Takara, Dalian, China) with a CFX Connect™ Real-Time PCR Detection System (BIO-RAD) by following the manufacturer's instructions. The specific primers of 20 *PbFRK* genes used for amplification were designed by the Beacon Designer 7 software (electronic supplementary material, table S6). In the present study, three biological replicates were carried out. The 2 ^–ΔΔCT^ method was used to calculate the relative expression level [58]. As the internal control gene, the *Pyrus* tubulin gene was selected according to previous studies [59].

## Supplementary Material

Figure S1.tif

## Supplementary Material

Figure S2.tif

## Supplementary Material

Table S1.xlsx

## Supplementary Material

Table S2.xlsx

## Supplementary Material

Table S3.xlsx

## Supplementary Material

Table S4.xlsx

## Supplementary Material

Table S5.xlsx

## Supplementary Material

Table S6.xlsx

## Data accessibility

Sequences of the here described genes and the accession numbers of publically available sequences that were used in this study are listed in table 1. We uploaded the experimental data as the electronic supplementary material.
