# Supplementary figures and images for: A new insight into the evolution and functional divergence of FRK genes in Pyrus bretschneideri
Source: R Soc Open Sci. 2018 Jul 18;5(7):171463. doi: 10.1098/rsos.171463 (PMC6083675; doi:10.1098/rsos.171463)

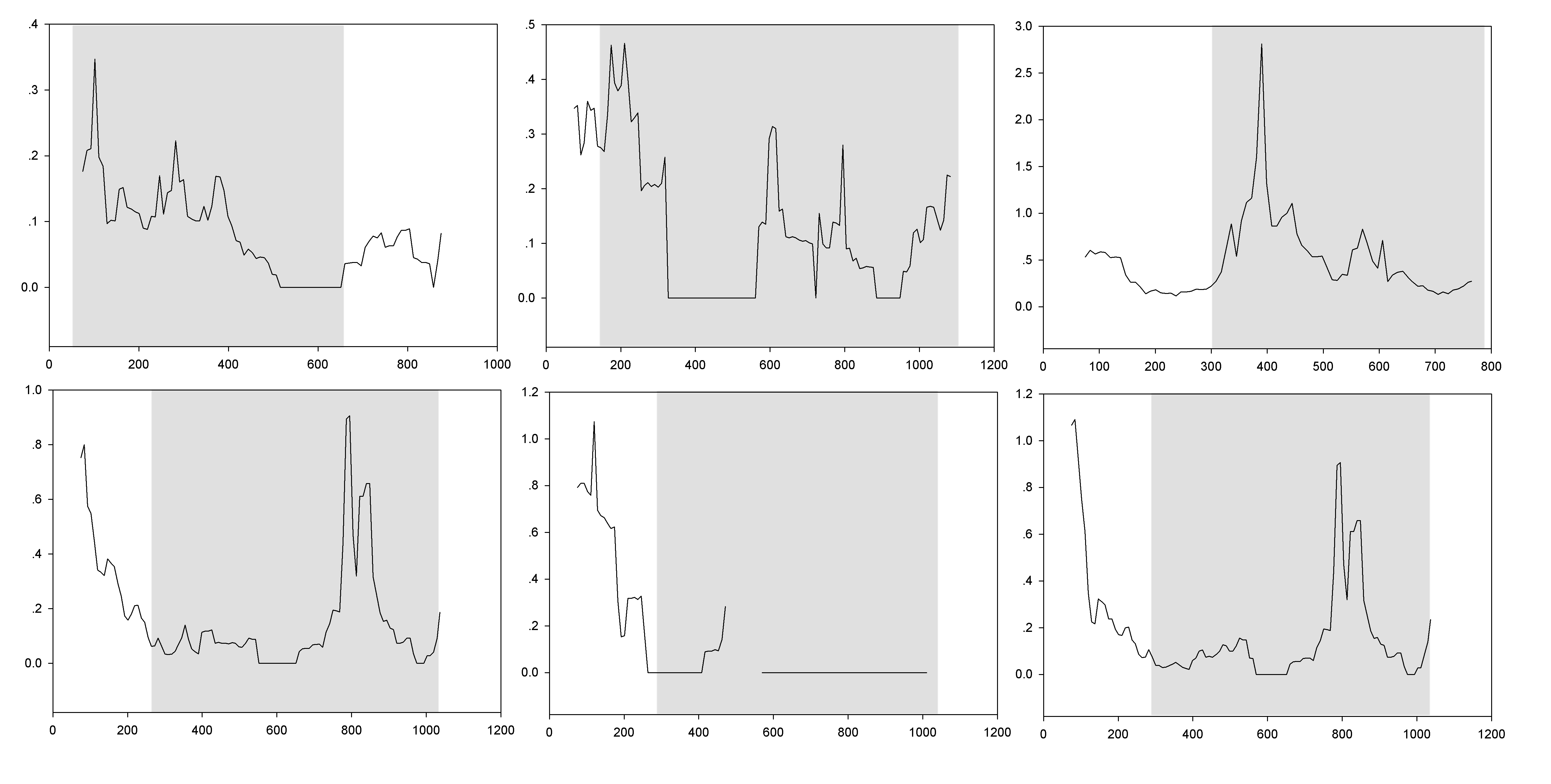

Supplement: Figure S1.tif [file rsos171463supp1.tif]

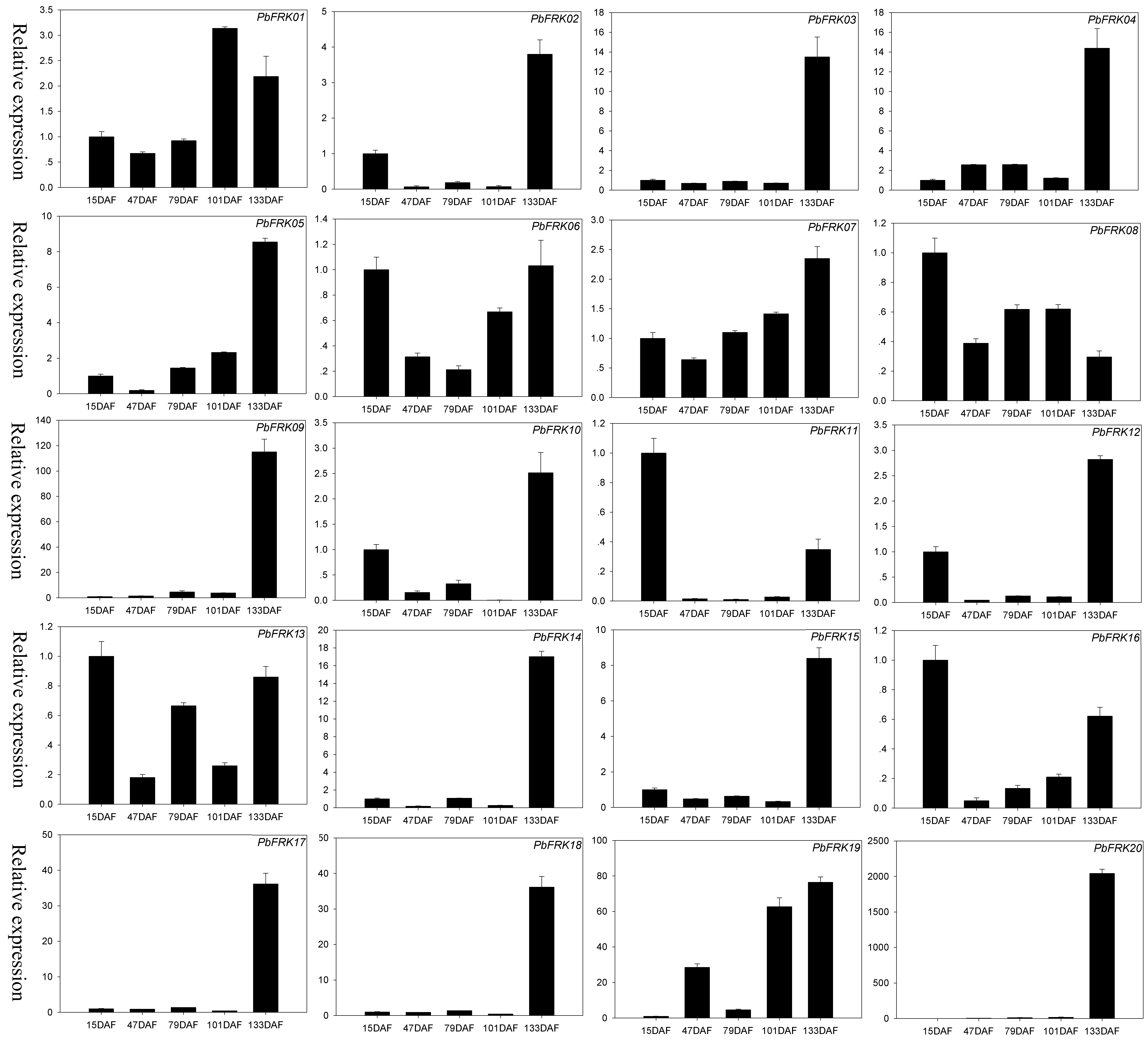

Supplement: Figure S2.tif [file rsos171463supp2.tif]
